# Supplementary material for: Morphologic determinant of tight junctions revealed by claudin-3 structures
Source: Nat Commun. 2019 Feb 18;10:816. doi: 10.1038/s41467-019-08760-7 (PMC6379431; doi:10.1038/s41467-019-08760-7)
Supplement: Supplementary file 1 — Supplementary Information [file 41467_2019_8760_MOESM1_ESM.pdf]

Supplementary Information

Morphologic determinant of tight junctions revealed by claudin-3 structures

S. Nakamura et al.

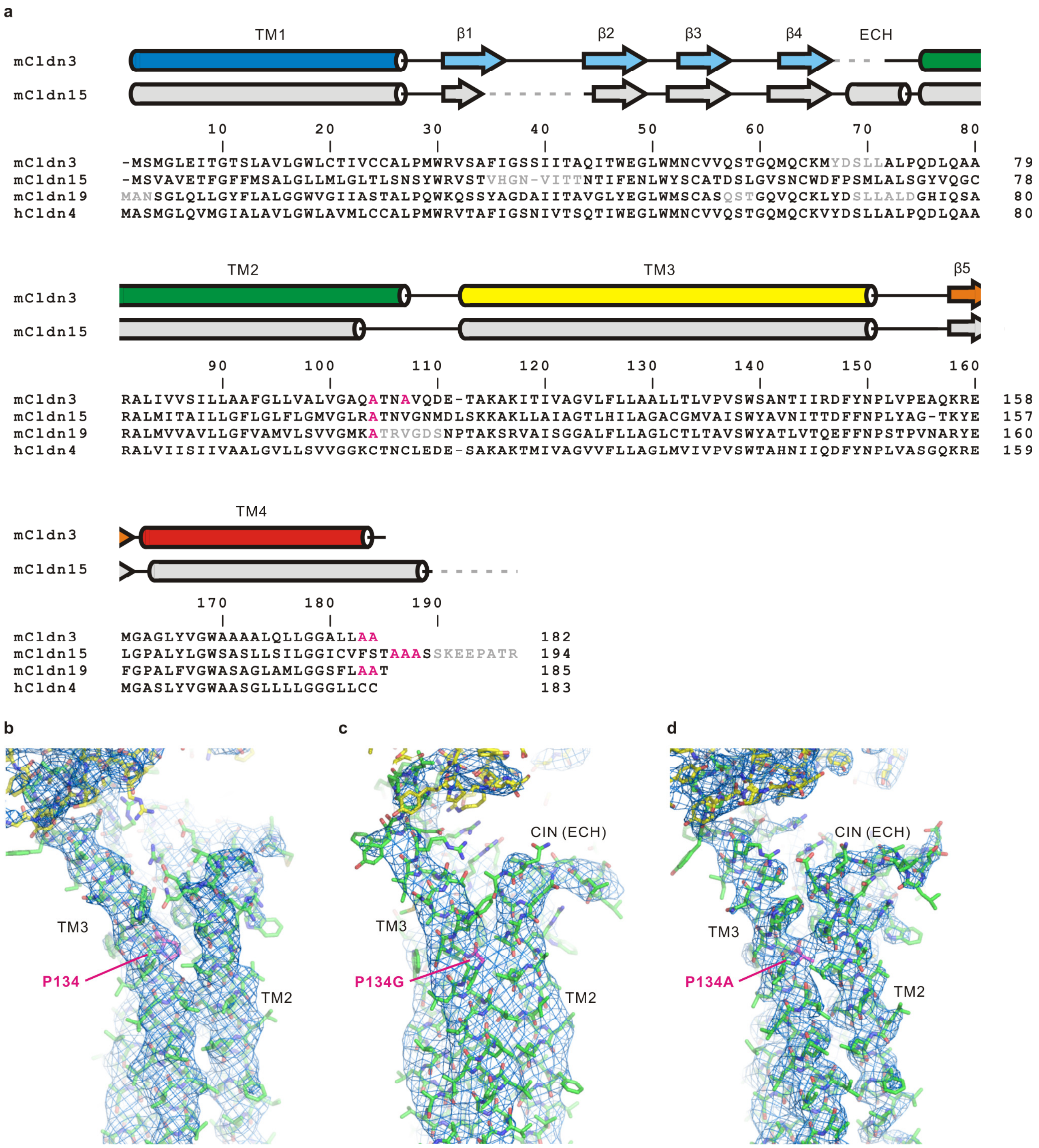

Supplementary Figure 1. Structure determination of mCldn3.

(a) Multiple sequence alignment of the following structure-determined Cldns; mCldn3$_{cryst}$, mCldn15 (PDB ID: 4p79), mCldn19 (PDB ID: 3x29), and hCldn4 (PDB ID: 5b2g). In the sequences, the cysteine residue into which the alanine mutation is introduced is indicated in magenta, and the disordered residue is indicated in gray. Schematic diagrams of the secondary structure of mCldn3 and mCldn15 are shown with α-helices and β-strands represented by cylinders and arrows, respectively. The disordered regions in the mCldn3$_{cryst}$ and mCldn15 are indicated by dashed lines. (b-d) Composite omit 2Fo-Fc electron density maps around the TM3 helix of mCldn3$_{cryst}$ (b), mCldn3$_{cryst}$ P134G (c) and mCldn3$_{cryst}$ P134A (d) at 3.6 Å, 4.3 Å and 3.9 Å, respectively, contoured at 2.0 σ (blue mesh). Final models are shown in stick representation, with mCldn3 colored green and C-CPE colored yellow. The residues that determine the TM3 structure are highlighted in magenta.

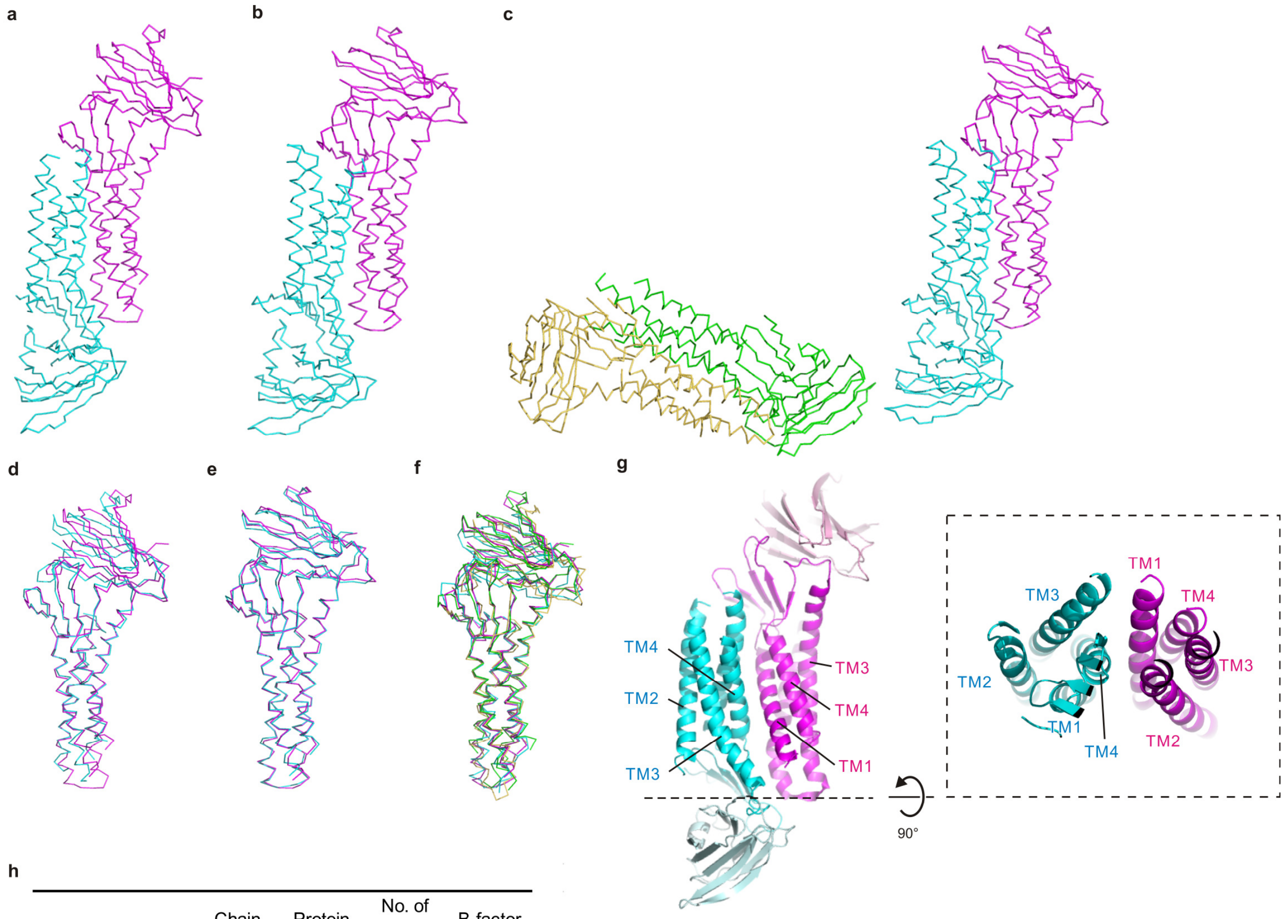

h

	Chain	Protein	No. of atoms	B-factor
mCldn3$_{cryst}$	A	mCldn3	1277	154.7
	B	C-CPE	918	161.9
	C	mCldn3	1210	156.7
	D	C-CPE	899	162.5
mCldn3$_{cryst}$ P134G	A	mCldn3	1323	245.7
	B	C-CPE	918	281.8
	C	mCldn3	1331	267.1
	D	C-CPE	910	303.9
mCldn3$_{cryst}$ P134A	A	mCldn3	1335	98.1
	B	C-CPE	925	86.7
	C	mCldn3	1350	104.1
	D	C-CPE	932	92.6
	E	mCldn3	1264	153.1
	F	C-CPE	928	141.5
	G	mCldn3	1222	151.4
	H	C-CPE	932	121.1

Supplementary Figure 2. mCldn3/C-CPE complexes in an asymmetric unit.
(a) Two mCldn3/C-CPE complexes in the asymmetric unit of mCldn3$_{cryst}$/C-CPE are shown in cyan (chain A/B) and magenta (chain C/D). (b) Two mCldn3/C-CPE complexes in the asymmetric unit of mCldn3$_{cryst}$ P134G/C-CPE are shown in cyan (chain A/B) and magenta (chain C/D), which contains one dimer of the mCldn3/C-CPE complex in the same manner as mCldn3$_{cryst}$. (c) Four mCldn3/C-CPE complexes in the asymmetric unit of mCldn3$_{cryst}$ P134A /C-CPE are shown in cyan (chain A/B), magenta (chain C/D), yellow (chain E/F), and green (chain G/H), which contains two dimers of the mCldn3/C-CPE complex. (d-f) Superposition of the complexes in the asymmetric unit of mCldn3$_{cryst}$ (d), mCldn3$_{cryst}$ P134G (e) and mCldn3$_{cryst}$ P134A (f). (g) Two complexes in the asymmetric unit of mCldn3$_{cryst}$/C-CPE in ribbon representation. The two complexes are bound by asymmetric interactions through the transmembrane region of mCldn3. (h) Number of atoms and B-factor.

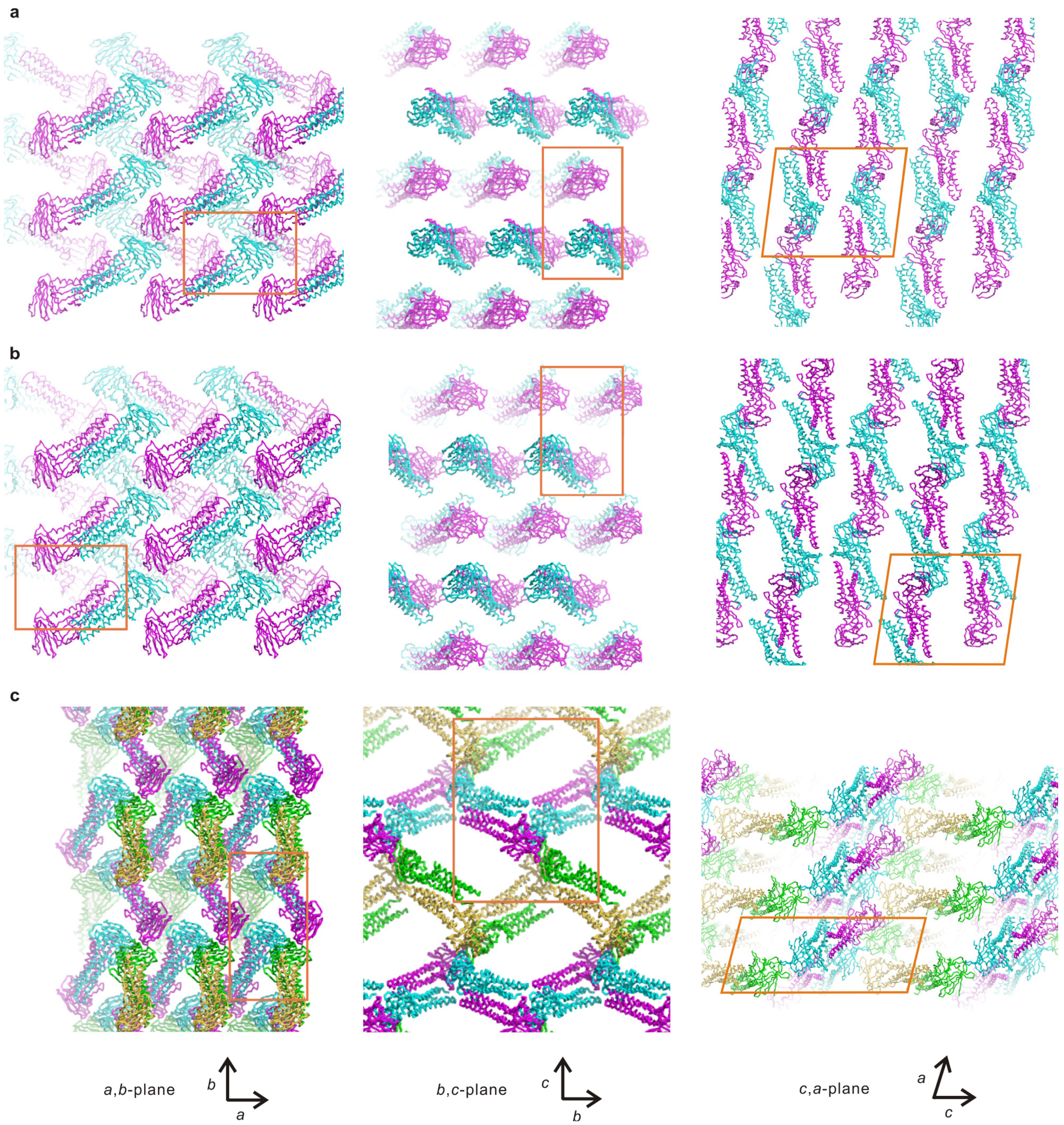

Supplementary Figure 3. Crystal packing.
Crystal packing of mCldn3$_{cryst}$/C-CPE (a), mCldn3$_{cryst}$ P134G/C-CPE (b) and mCldn3$_{cryst}$ P134A/C-CPE (c), viewed perpendicular to the *a*,*b*-plane (left), *b*,*c*-plane (middle), and *c*,*a*-plane (right). Orange square indicates the unit cell.

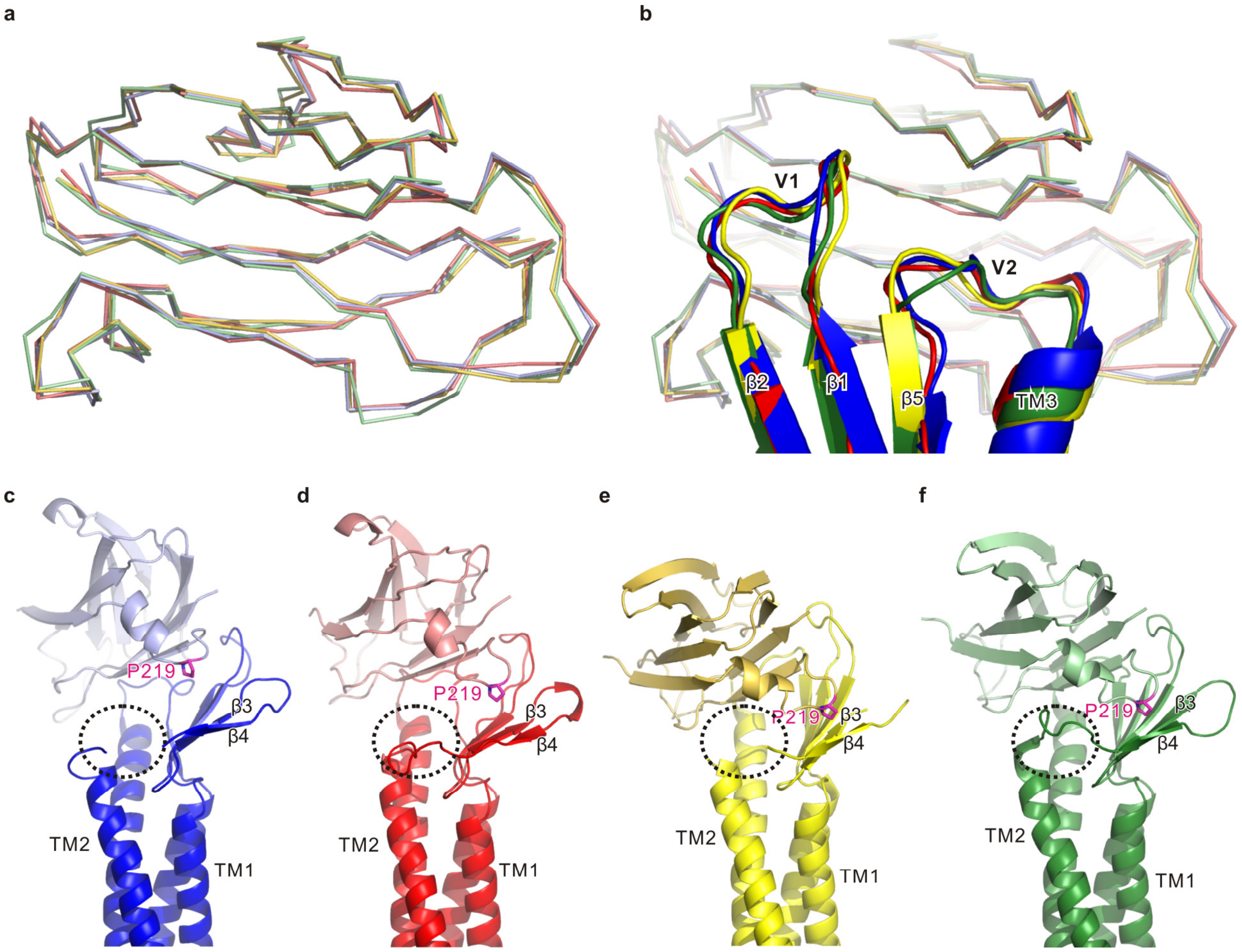

Supplementary Figure 4. C-CPE binding.
(a) Superposition of C-CPEs paired with $mCldn3_{cryst}$, $mCldn3_{cryst}$ P134A, mCldn19, and hCldn4. C-CPEs paired with $mCldn3_{cryst}$, $mCldn3_{cryst}$ P134A, mCldn19, and hCldn4 are colored light blue, salmon, light yellow, and pale green, respectively. (b) Superposition of V1 and V2 region of $mCldn3_{cryst}$ (blue), $mCldn3_{cryst}$ P134A (red), mCldn19 (yellow), and hCldn4 (green), based on the C-CPE structures. (c and d) In the structures of $mCldn3_{cryst}$ (c) and $mCldn3_{cryst}$ P134A (d), C-CPE binds to mCldn3 apart from the β sheet. (e and f) In the mCldn19 (e) and hCldn4 (f) structures, C-CPE binds to mCldn19 and hCldn4 close to the β sheet. Pro219 on C-CPE, closest to the β sheet, is shown in magenta stick representation. The region corresponding to the ECH is indicated by the dotted circle.

a

```
                        CIN (ECH)
                    nnn                   nnn
mCldn3      65  MYDSL---------------------LALPQ   74
mCldn15     64  DFPSM---------------------LALSG   73
mCldn1      66  VFDSL---------------------LNLNS   75
mCldn2      66  IYSTL---------------------LGLPA   75
mCldn4      66  MYDSM---------------------LALPQ   75
mCldn5      66  VYESV---------------------LALSA   75
mCldn6      66  VYDSL---------------------LALPQ   75
mCldn7      66  MYDSV---------------------LALPG   75
mCldn8      66  VYDSL---------------------LALSP   75
mCldn9      66  VYDSL---------------------LALPQ   75
mCldn10a    63  PHFTI---------------------FKVEG   72
mCldn10b    65  EFPSM---------------------LALDG   74
mCldn11     67  PLVDI---------------------LILPG   76
mCldn12     69  MYDRTWY------------------LSVDQL   81
mCldn13     65  LYDTR---------------------ILVAQ   74
mCldn14     66  IYRSL---------------------LALPR   75
mCldn16     63  EYDSIY--------------------AEHPL   73
mCldn17     66  FYNSI---------------------LALPP   75
mCldn18lg   65  PYFTI---------------------LGLPA   74
mCldn18st   65  GYFTL---------------------LGLPA   74
mCldn19     66  LYDSL---------------------LALDG   75
mCldn20     66  LKYSI---------------------LSLPV   75
mCldn21     66  AFESF---------------------LSLPQ   75
mCldn22     66  DFDSF---------------------LALPA   75
mCldn23     65  QPDEWN--------------------YFQTQ   75
mCldn24     66  DFDSF---------------------LALPA   75
mCldn25     93  PPERTESFDVVTKCMSFTLNEQFMEKYVDPG  123
mCldn26     83  PFWQEN--------------------VTVSD   93
mCldn27     69  PFASAG--------------------LEVSP   79
```

b

```
                       TM3                                      β5
                        *           pp                                  pp
mCldn3     126  LAALLTLVPVSWSANTIIRDFYN--------------PLVPEAQKREMGA  161
mCldn15    126  LAGACGMVAISWYAVNITTDFFN---------------PLYAGTKYELGP  160
mCldn1     128  ISGLATLVATAWYGNRIVQEFYD--------------PLTPINARYEFGQ  163
mCldn2     127  LGGILGFIPVAWNLHGILRDFYS--------------PLVPDSMKFEIGE  162
mCldn4     127  VASMLIMVPVSWTAHNVIRDFYN--------------PMVASGQKREMGA  162
mCldn5     127  VCGLLALVPLCWFANIVVREFYD--------------PTVPVSQKYELGA  162
mCldn6     127  ISGVLTLIPVCWTAHSIIQDFYN--------------PLVADAQKRELGA  162
mCldn7     128  VAGLAALVACSWIGHQIVTDFYN--------------PLTPMNVKYEFGP  163
mCldn8     128  ITGLVVLIPVSWVANSIIRDFYN--------------PLVDVALKRELGE  163
mCldn9     127  LSGILVLIPVCWTAHAIIQDFYN--------------PLVAEALKRELGA  162
mCldn10a   124  LSGLCSMTGCSLYANKITTEFFD---------------PLYMEQKYELGA  158
mCldn10b   126  LSGLCSMTGCSLYANKITTEFFD---------------PLYMEQKYELGA  160
mCldn11    129  LLALCAIVATIWFP----------------------VCAHREITIVSFGY  155
mCldn12    136  AGLLFFLAGTVSLSPSIWAIFYN------------SHLNRKFEPVFTFDY  173
mCldn13    126  SVGLLMLVPLSWVTHNIIHGFFN--------------PLLGFSKKVQMGS  161
mCldn14    127  LAGLLCMVAVSWTTNDVVQNFYN--------------PLLPSGMKFEIGQ  162
mCldn16    126  IAGTPGIIGSVWYAVDVYVERSS-----------LVLHNIFLGIQYKFGW  164
mCldn17    128  LTGIFVLIPVSWTANIIIRDFYD--------------PTVHAGQKRELGG  163
mCldn18lg  127  ISGICAIIGVSVFANMLVTNFWMSTANMYSGMGGMGGMVQTVQTRYTFGA  176
mCldn18st  127  ISGICAIIGVSVFANMLVTNFWMSTANMYSGMGGMGGMVQTVQTRYTFGA  176
mCldn19    128  LAGLCTLTAVSWYATLVTQEFFN--------------PSTPVNARYEFGP  163
mCldn20    127  TAGISGLIPTVWYTKEIIANFLD--------------LTIPESHKYEPGG  162
mCldn21    128  SASATTLFPVSWVAYATFQDFWD-------------DSIPEIVPRWEFGD  164
mCldn22    128  TSGVMVLVPVSWVAHKTVREFWD-------------ETMPEIVPRWEFGE  164
mCldn23    123  VAGLFSLIPVSWYNHFLSDPDVL--------------AAPSSPVTVQVSY  158
mCldn24    128  TSGVMVLVPVSWVAHKTVREFWD-------------ETMPEIVPRWEFGE  164
mCldn25    179  LAGLCTLGSVSCYVAGIELLHQK--------------VELPKDVSGEFGW  214
mCldn26    144  FGAMVTLTGISIYIAYSAVAFREAVC-------LLEERALLDQVDIRFGW  186
mCldn27    130  LGGALTLAGLSIYISYSHLAFVEAAR-------TYGVTHVQN-VHISFGW  171
```

Supplementary Figure 5. Multiple sequence alignment among mouse Cldn family. Protein sequences of the CIN (previously ECH) periphery (a) and TM3-β5 region (b) in the following mouse Cldn subtypes are aligned; mCldn1 (RefSeq ID: NP_057883.1), mCldn2 (NP_057884.1), mCldn3 (NP_034032.1), mCldn4 (NP_034033.1), mCldn5 (NP_038833.2), mCldn6 (NP_061247.1), mCldn7 (NP_001180548.1), mCldn8 (NP_061248.1), mCldn9

(NP_064689.2), mCldn10a (NP_076367.2), mCldn10b (NP_067361.2), mCldn11 (NP_0.32796.1), mCldn12 (NP_075028.1), mCldn13 (NP_065250.1), mCldn14 (NP_001159397.1), mCldn15 (NP_068365.1), mCldn16 (NP_444471.1), mCldn17 (NP_852467.1), mCldn18lg (NP_062789.1), mCldn18st (NP_001181850.1), mCldn19 (NP_694745.1), mCldn20 (NP_001095030.1), mCldn21 (XP_001478861.1), mCldn22 (NP_083659.1), mCldn23 (NP_082274.1), mCldn24 (NP_001104788.1), mCldn25 (NP_741968.1), mCldn26 (NP_083346.1), mCldn27 (NP_001079004.1). Asterisk above the sequences indicates the residue determining the TM3 structure, and "n" and "p" indicate residues constituting the CIN (ECH) and cis-interaction pocket, respectively. Proline, glycine, and alanine are indicated in black, olive, and cyan, respectively. Among hydrophobic amino acids, aromatic amino acids are indicated in deep green, others in lighter green. Hydrophilic amino acids are indicated in yellow, acidic amino acids in red, and basic amino acids in blue.

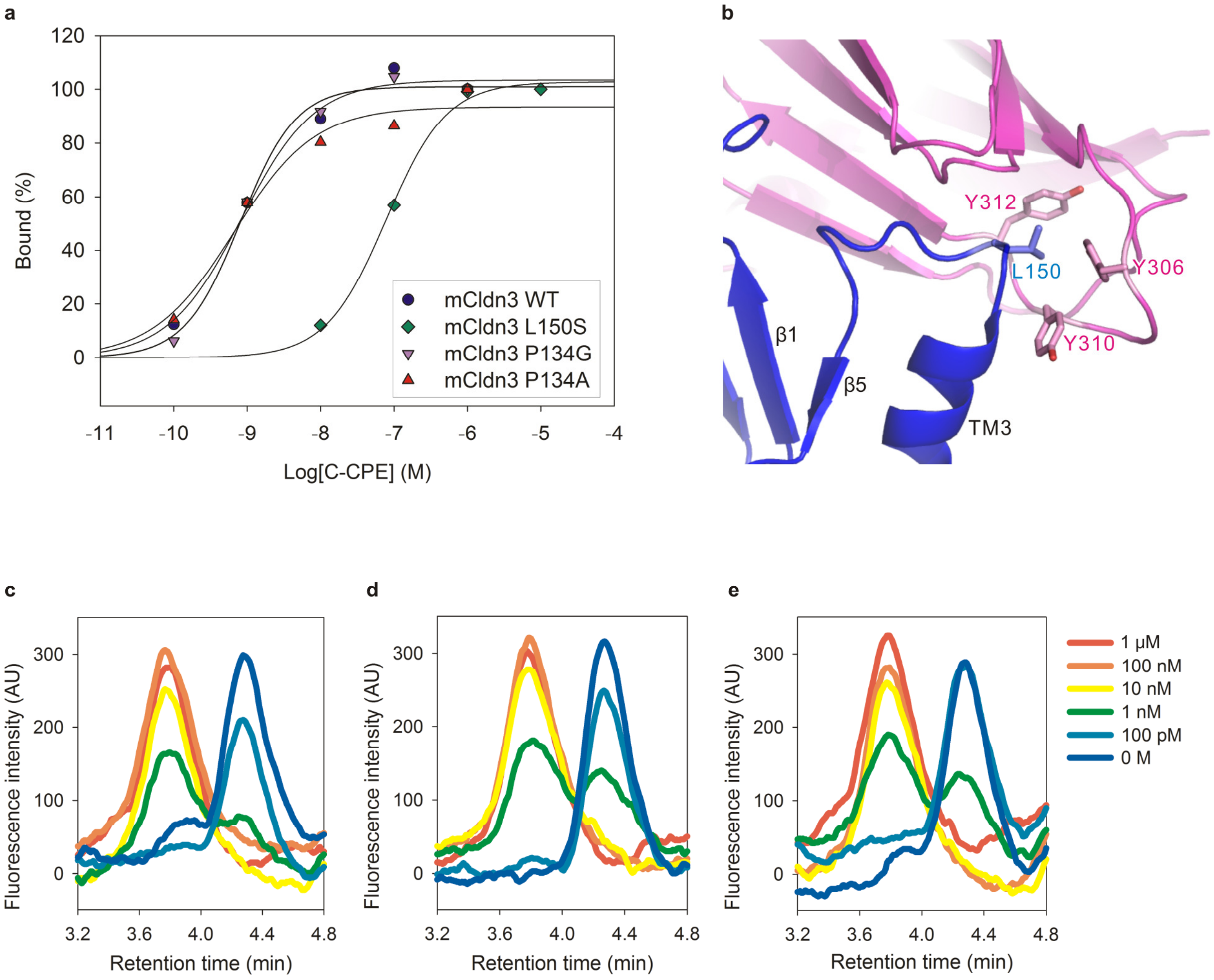

Supplementary Figure 6. Binding affinity of mCldn3 for C-CPE.
(a) Dose-response curves of mCldn3 WT and mutants. (b) A key residue for C-CPE affinity. mCldn3 and C-CPE are colored blue and magenta, respectively. (c-e) Chromatograms of FSEC analyses. EGFP-fused mCldn3 wild-type (WT) (c), P134G mutant (d) and P134A mutant (e) are incubated with different concentrations (0-1 μM) of GST-C-CPE, and analyzed using FSEC by detecting EGFP fluorescence (excitation wavelength = 488 nm, emission wavelength = 509 nm). Peaks at a retention time of 3.8 min and 4.3 min are derived from the EGFP-mCldn3/GST-C-CPE complex and EGFP-mCldn3, respectively.

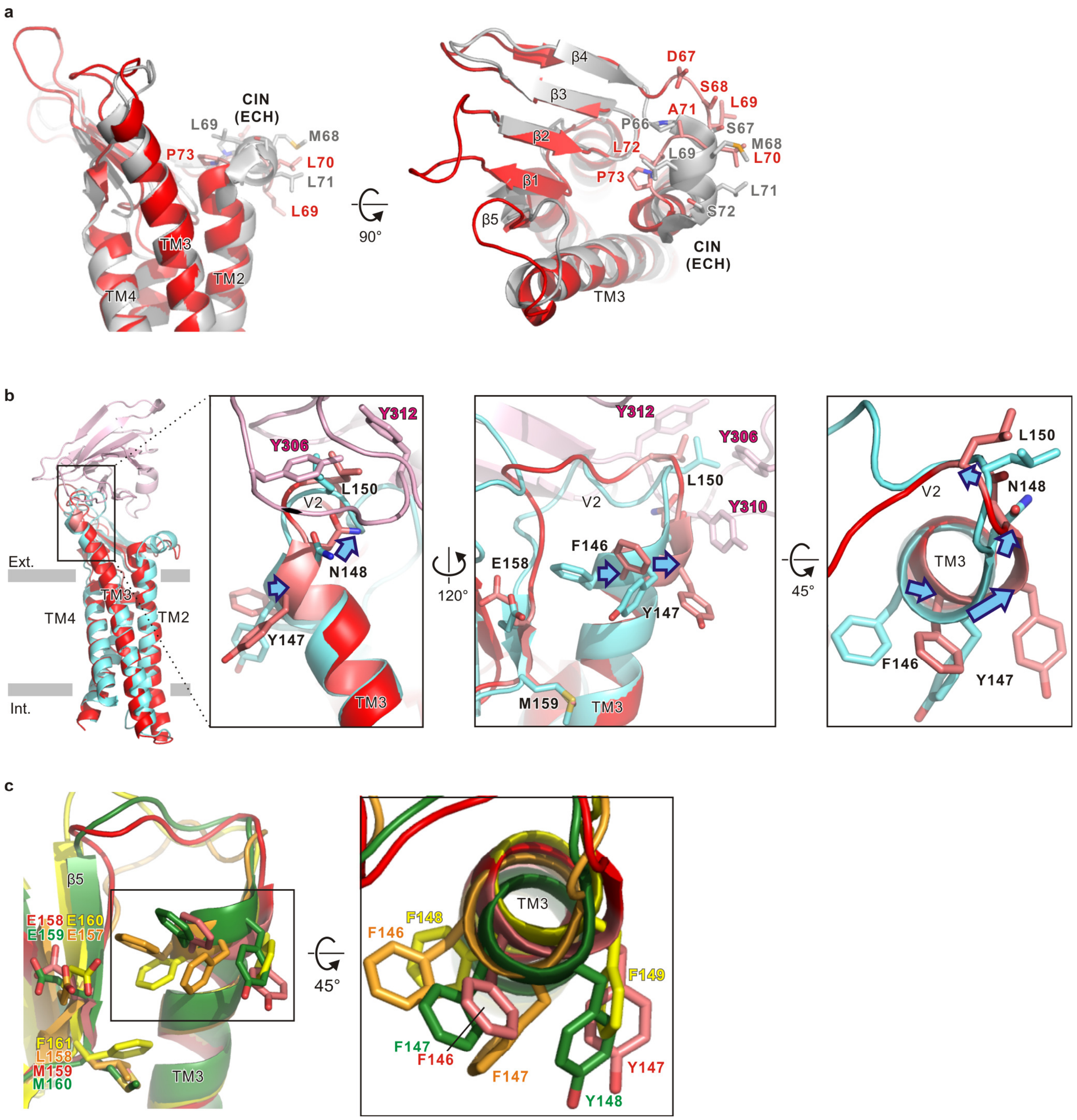

Supplementary Figure 7. Influence of C-CPE binding on Cldn structure.
(a) Structure of the CIN (ECH) periphery in mCldn3 P134A mutant (red) and mCldn15 (gray). (b) Structural comparison of the mCldn3 homology model in the C-CPE unbound state (cyan) and the mCldn3 P134A mutant in the C-CPE bound state (red) shows disruption of the cis-interaction pocket by C-CPE binding. C-CPE paired with the P134A mutant is colored pink. Blue arrows indicate the direction of the structural change induced by C-CPE binding. Residue names of mCldn3 and C-CPE are colored black and pink, respectively. (c) mCldn3 P134A mutant (C-CPE bound), mCldn15 (C-CPE unbound), mCldn19 (C-CPE bound), and hCldn4 (C-CPE bound) are indicated by red, orange, yellow, and green. The residues constituting the cis-interaction pocket are shown in stick representation.

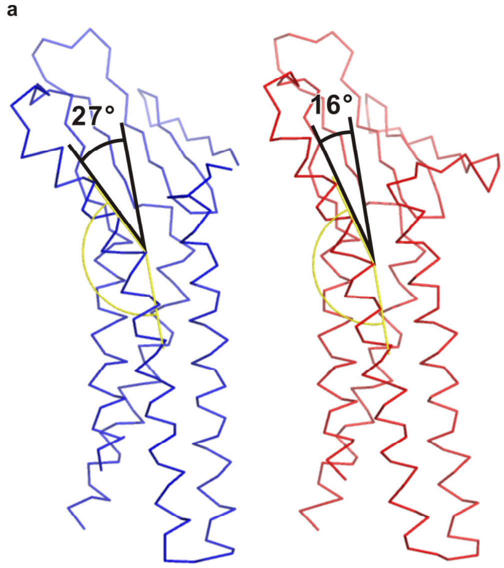

Subtype	TM3†	Chain‡	Angle (°) Individual	Angle (°) mean
mCldn3	P134	A	27	25
		C	23	
	P134 A	A	16	17
		C	19	
		E	16	
		G	19	
	P134 G	A	17	17
		C	16	
hCldn4	P135	A	19	20
		C	19	
		E	21	
		G	21	
mCldn15	A134	-	16	16
mCldn19	A136	A	14	13
		C	12	

† The residue determining TM3 structure.
‡ Angles were calculated for all molecular chains in the asymmetric unit.

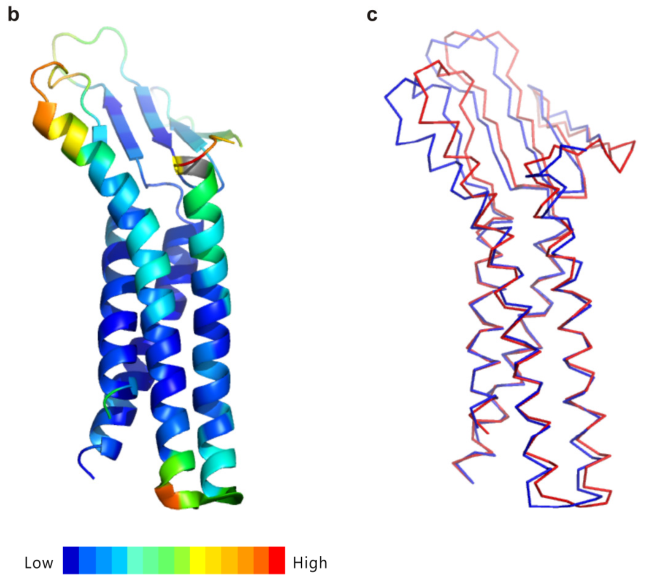

Supplementary Figure 8. Comparison between mCldn3$_{cryst}$ and mCldn3$_{cryst}$ P134A.
(a) Angles of TM3 bending were calculated as follows; define Cα of Pro134 on mCldn3 or the corresponding residue as the vertex of the angle; as another two atoms forming the angle, select the nitrogen atom of the main chain of the 7th residue on the N terminal side from Pro134 and the carbonyl carbon atom of the main chain of the 7th residue on the C terminal side from Pro134; calculate the angle formed by connecting the vertex and the two atoms using PyMOL (yellow angle); then, the angles of TM3 bending were calculated for all structure-determined subtypes, shown in the above table. Cα chains of mCldn3$_{cryst}$ and mCldn3$_{cryst}$ P134A are indicated by blue and red, respectively. Regarding the two molecules in the asymmetric unit of mCldn3$_{cryst}$, chains A and C, the bending angle of chain A is slightly larger than that of chain C. In the crystal packing, chain A has no steric hindrance in the direction of TM3 bending, whereas the TM3 of chain C is hampered bending by TM1 of chain A (Supplementary Figure 2g). Therefore, it is suggested that the structure with TM3 largely bending like chain A is nearer to the native conformation. (b) Distances between aligned Cα atom pairs between mCldn3$_{cryst}$ and mCldn3$_{cryst}$ P134A are colored on the mCldn3$_{cryst}$ structure by a color spectrum, with blue indicating the minimum pairwise RMSD (0.09 Å) and red indicating the maximum (5.38 Å). (c) Superposition based on a low RMSD region in TMD between mCldn3$_{cryst}$ and mCldn3$_{cryst}$ P134A. mCldn3$_{cryst}$ and mCldn3$_{cryst}$ P134A are colored blue and red, respectively.

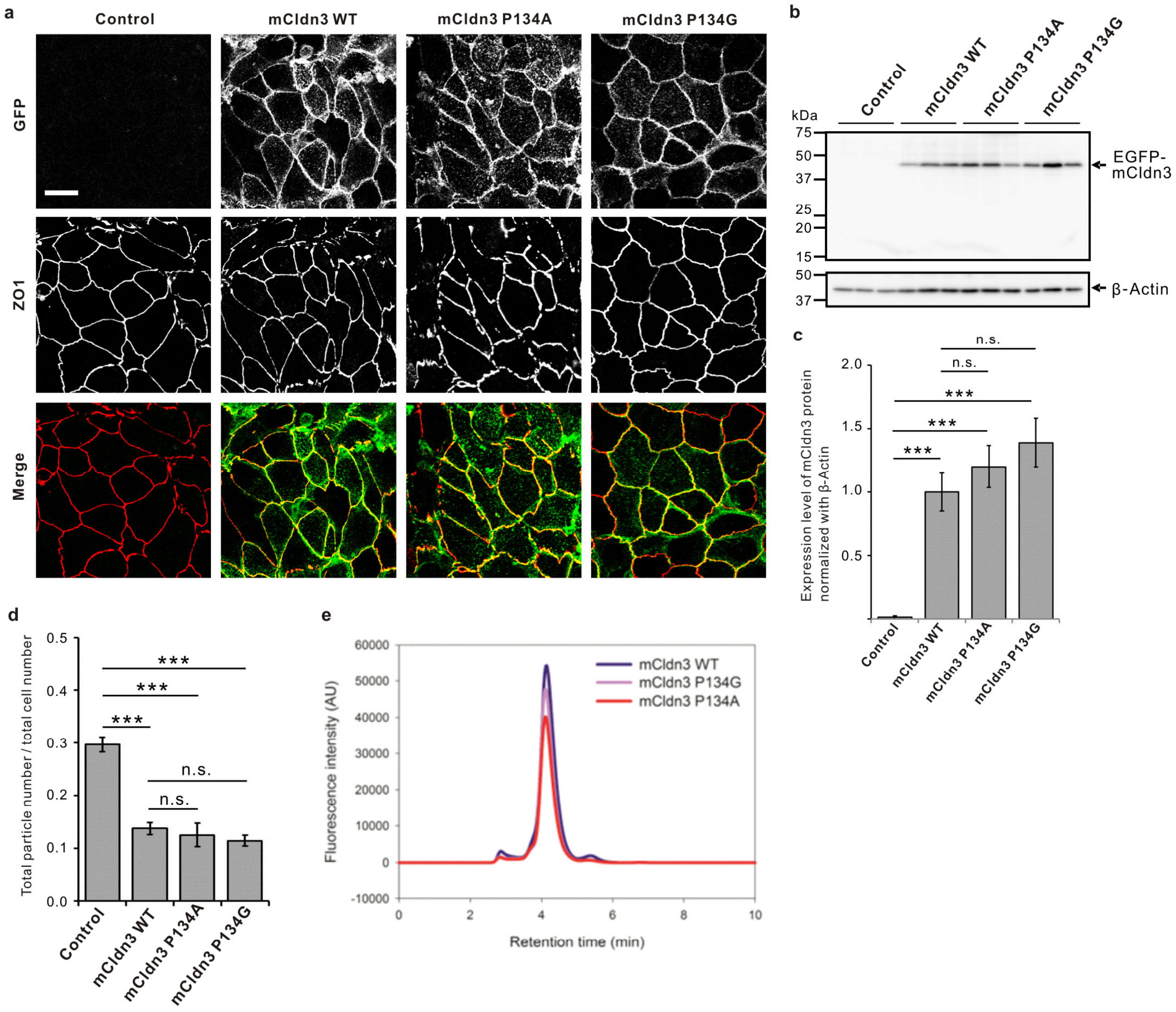

Supplementary Figure 9. Expression state of mCldn3 on mammalian cells.
(a) Immunofluorescence microscopy of parental SF7 cells (control), and stably expressing EGFP-fused mCldn3 (mCldn3 WT, P134A, or P134G). Immunofluorescence staining for GFP (green) with co-staining for ZO1 (red) is shown. Bar, 10 µm. (b) Western blotting shows the expression levels of mCldn3 in SF7-derived cell lines (control, mCldn3 WT, P134A, or P134G). Results from three distinct clones are shown. (c) The expression levels of mCldn3 in SF7-derived cell lines (control, mCldn3 WT, P134A, or P134G) detected by Western blotting are normalized with β-actin (n=3/group). Results from three distinct clones are shown as means ± SEM. P-values were calculated using a two-tailed independent t-test, and P < 0.05 was considered significant. n.s., not significant; ***, $p < 0.001$. (d) Dissociation assay under 8-pipetting conditions shows that SF7 cells stably expressing EGFP-fused mCldn3 (mCldn3 WT, P134A, or P134G) (n=9/group) form TJs because of stronger adhesion than the parental SF7 cells (control) (n=7). Results from three distinct clones are shown as means ± SEM. P-values were calculated using a two-tailed independent t-test, and P < 0.05 was considered significant. n.s., not significant; ***, p<0.001. (e) HEK293 cells expressing EGFP-fused mCldn3 were solubilized and analyzed using FSEC by detecting EGFP fluorescence (excitation wavelength = 488 nm, emission wavelength = 509 nm). Chromatograms of mCldn3 WT, P134G, and P134A are shown in blue, violet, and red, respectively. Peaks at a retention time of 4.1 min are derived from EGFP-mCldn3. Source data are provided as a Source Data file (b, c, d).

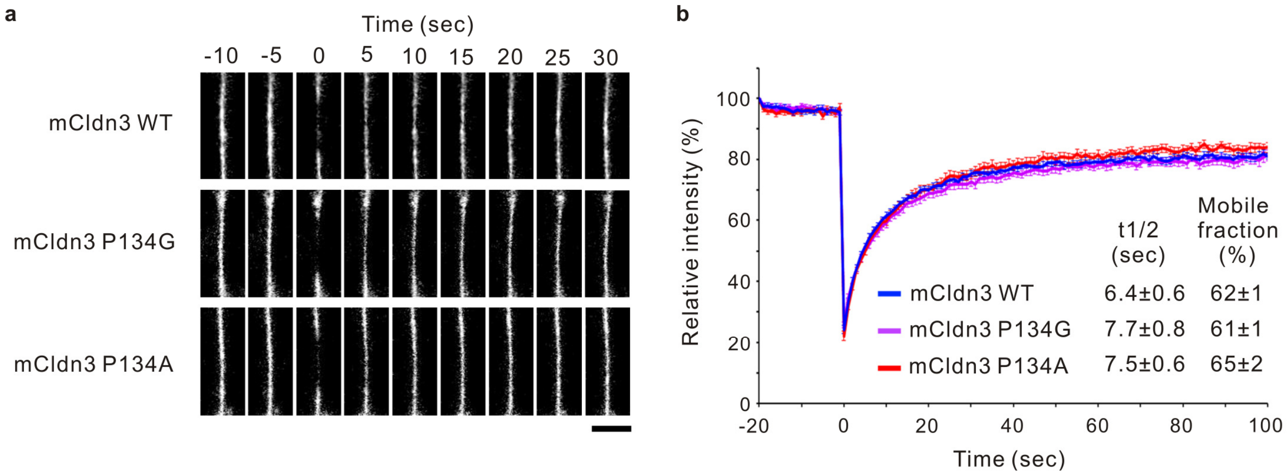

Supplementary Figure 10. FRAP dynamics of mCldn3 on mammalian cells

(a) FRAP analysis of EGFP-fused mCldn3 (mCldn3 WT, P134G or P134A) stably expressed in SF7 cells. Representative high-magnification images of TJ segments at the indicated time points after photobleaching are shown. Bar, 5 µm. (b) Quantification of FRAP analysis of EGFP-fused mCldn3 (mCldn3 WT, P134G or P134A) stably expressed in SF7 cells. The half-time (t1/2) of fluorescence recovery for mCldn3 WT, P134G or P134A are 6.4±0.6, 7.7±0.8, or 7.5±0.6 sec, respectively. The mobile fractions for mCldn3 WT, P134G or P134A are 62±1, 61±1, or 65±2 %, respectively. The t1/2 of fluorescence recovery and mobile fractions show no significant differences among mCldn3 WT, P134G and P134A. Results from three distinct clones are shown as means ± SEM (n ≥15/group). P-values were calculated using a two-tailed independent t-test, and $P < 0.05$ was considered significant. Source data are provided as a Source Data file (b).

Supplementary Table 1. Crystallographic data collection and refinement statistics.

	**mCldn3$_{cryst}$ with C-CPE**	**mCldn3$_{cryst}$ P134G with C-CPE**	**mCldn3$_{cryst}$ P134A with C-CPE**
**Data collection**			
Resolution (Å)	3.6×3.6×3.9 † (3.7 - 3.6) ‡	4.3×4.3×5.3 † (4.4 - 4.3) ‡	3.9 (4.0 - 3.9) ‡
Space group	$P\ 1\ 2_1\ 1$	$P\ 1\ 2_1\ 1$	$P\ 1\ 2_1\ 1$
Cell dimensions			
a, b, c (Å)	92.6 66.63 111.54	91.71 68.21 107.88	69.54 127.45 165.7
α, β, γ (˚)	90 97.55 90	90 98.7 90	90 104.53 90
R_{merge}	0.048 (1.190)	0.039 (1.827)	0.091 (1.032)
R_{pim}	0.024 (0.583)	0.016 (0.739)	0.059 (0.682)
CC1/2	0.999 (0.561)	0.999 (0.689)	0.998 (0.575)
Mean $I/\sigma I$	14.86 (1.51)	14.82 (1.31)	8.17 (1.32)
Completeness (%)	93.92 (60.68)	88.85 (53.84)	98.50 (99.49)
Redundancy	5.0 (5.3)	6.7 (7.0)	3.3 (3.3)
**Refinement**			
Resolution (Å)	45.9 - 3.6 (3.7 - 3.6)	49.3 - 4.3 (4.4 - 4.3)	46.6 - 3.9 (4.0 - 3.9)
No. of reflections	15888 (952)	9133 (490)	25301 (2529)
R_{work}/ R_{free} (%)	25.4/28.7 (41.9/38.1)	28.3/32.9 (32.2/37.4)	27.4/31.2 (35.9/38.3)
Wilson B-factor	147.29	214.53	95.56
No. of atoms	4304	4482	8888
Average B-factor	158.44	271.26	119.35
R.m.s deviations			
Bond lengths (Å)	0.012	0.014	0.011
Bond angles (˚)	1.54	1.66	1.54
Ramachandran favoured (%)	98.40	98.48	98.18
allowed (%)	1.60	1.52	1.82
outliers (%)	0.00	0.00	0.00

† The diffraction data are anisotropic. The resolution limits here are for the *a**, *b** and *c** axes, respectively.

‡ Statistics for the highest-resolution shell are shown in parentheses.

Supplementary Table 2. Primer list

Name	Sequence
GFP-mCldn3_for_GibsonAssembly_Forward	AAGAAGGAGATATACCATGGTGAGCAAGGGCGAGGAGC
GFP-mCldn3_for_GibsonAssembly_Reverse	CGAACCGGTACCGTCGACTCAGACGTAGTCCTTGCGGTC
mCldn3_P134G_Forward	CTTAGTAGGGGTGTCCTGGTCGGCCAAC
mCldn3_P134G_Reverse	GGACACCCCTACTAAGGTGAGCAGAGC
mCldn3_P134A_Forward	CTTAGTAGCGGTGTCCTGGTCGGCCAAC
mCldn3_P134A_Reverse	GGACACCGCTACTAAGGTGAGCAGAGC
mCldn3_L150S_Forward	TAACCCGTCCGTGCCCGAGGCCCAGAAGC
mCldn3_L150S_Reverse	CGGGCACGGACGGGTTATAGAAATCCCTG
mCldn3_for_BacMam_Forward	TAGCGAATTCGTCGACATGTCCATGGGCCTGGAGATCA
mCldn3_for_BacMam_Reverse	CCGCGCATGCCTCGAGTCAGACGTAGTCCTTGCGGT
